# Munc18c in Adipose Tissue Is Downregulated in Obesity and Is Associated with Insulin

**DOI:** 10.1371/journal.pone.0063937

**Published:** 2013-05-20

**Authors:** Lourdes Garrido-Sanchez, Xavier Escote, Leticia Coin-Aragüez, Jose Carlos Fernandez-Garcia, Rajaa El Bekay, Joan Vendrell, Eduardo Garcia-Fuentes, Francisco J. Tinahones

**Affiliations:** 1 Endocrinology and Diabetes Unit, Joan XXIII University Hospital, IISPV, Universitat Rovira i Virgili, Tarragona, Spain; 2 CIBER de Diabetes y Enfermedades Metabólicas Asociadas (CIBERDEM), Instituto de Salud Carlos III, Tarragona, Spain; 3 Servicio de Endocrinología y Nutrición, Hospital Clínico Virgen de la Victoria, Malaga, Spain; 4 Ciber Fisiopatología Obesidad y Nutrición (CIBEROBN), Instituto de Salud Carlos III, Málaga, Spain; 5 Instituto de Investigaciones Biomédicas de Málaga (IBIMA), Málaga, Spain; 6 Servicio de Endocrinología y Nutrición, Hospital Regional Universitario Carlos Haya, Malaga, Spain; Tohoku University, Japan

## Abstract

**Objective:**

Munc18c is associated with glucose metabolism and could play a relevant role in obesity. However, little is known about the regulation of Munc18c expression. We analyzed Munc18c gene expression in human visceral (VAT) and subcutaneous (SAT) adipose tissue and its relationship with obesity and insulin.

**Materials and Methods:**

We evaluated 70 subjects distributed in 12 non-obese lean subjects, 23 overweight subjects, 12 obese subjects and 23 nondiabetic morbidly obese patients (11 with low insulin resistance and 12 with high insulin resistance).

**Results:**

The lean, overweight and obese persons had a greater Munc18c gene expression in adipose tissue than the morbidly obese patients (p<0.001). VAT Munc18c gene expression was predicted by the body mass index (B = −0.001, *p* = 0.009). In SAT, no associations were found by different multiple regression analysis models. SAT Munc18c gene expression was the main determinant of the improvement in the HOMA-IR index 15 days after bariatric surgery (B = −2148.4, *p* = 0.038). SAT explant cultures showed that insulin produced a significant down-regulation of Munc18c gene expression (p = 0.048). This decrease was also obtained when explants were incubated with liver X receptor alpha (LXRα) agonist, either without (p = 0.038) or with insulin (p = 0.050). However, Munc18c gene expression was not affected when explants were incubated with insulin plus a sterol regulatory element-binding protein-1c (SREBP-1c) inhibitor (p = 0.504).

**Conclusions:**

Munc18c gene expression in human adipose tissue is down-regulated in morbid obesity. Insulin may have an effect on the Munc18c expression, probably through LXRα and SREBP-1c.

## Introduction

Obesity has been considered to be associated with a proinflammatory state, generating an increased incidence of diabetes [1]. It has been well established that insulin stimulates glucose uptake in adipose tissue through the translocation of glucose transport protein 4 (GLUT4)-containing vesicles from intracellular storage sites to the plasma membrane [2]–[6]. This ultimately results in a large increase in the number of functional glucose transporters on the cell surface. The insulin-stimulated translocation of GLUT4-containing vesicles is a complex multistep process [4]–[6]. Numerous proteins are involved in the vesicular transport of GLUT4 [7]–[10]. Some of these proteins appear to regulate the assembly of these vesicles, such as syntaxin-binding protein 3 (STXBP3 or Munc18c) [11]. This protein is a mammalian homolog of two regulators of vesicle trafficking in *Saccharomycess cerevisiae* (Sec1) and *Caenorhabditis elegans* (UNC-18). Three human isoforms of Munc18 have been identified: Munc18-1 (Munc18a), Munc18b and Munc18c [11]–[13]. Munc18a is expressed in neuronal tissue [13], [14], whereas Munc18b and Munc18c are expressed ubiquitously, though only Munc18c is involved in the regulation of GLUT4 translocation in adipocytes [11], [15]–[17]. These proteins bind with high affinity to their cognate plasma membrane syntaxins, and null mutations in these genes cause reduction in vesicle exocytosis [18], [19]. This protein family is thought to play important roles in membrane trafficking and membrane fusion reactions [20].

The function of Munc18c in insulin-stimulated exocytosis of GLUT4-containing vesicles in adipose tissue is unclear. Different studies have suggested that overexpression of Munc18c plays an inhibitory role in insulin-stimulated GLUT4 translocation to the plasma membrane [15], [16], [21]. Other studies suggest that Munc18c is required for the insulin-induced fusion of GLUT4 vesicles with the plasma membrane [17]. The reduction of Munc18c protein in Munc18c^−/+^ mice results in impaired insulin sensitivity with a latent increased susceptibility for developing severe glucose intolerance [22]. These studies suggest a possible regulatory role of Munc18c in GLUT4 translocation and glucose transport. Munc18c is known to be phosphorylated on tyrosine-521 upon insulin stimulation of 3T3-L1 adipocytes [23], [24], [25]. This phosphorylation impairs the ability of Munc18c to bind its cognate soluble N-ethylmaleimide-sensitive factor attachment protein receptor (SNARE) proteins and may therefore represent a regulatory step in GLUT4 traffic [26]. These studies suggest that Munc18c can be regulated by insulin. However, little is known about the regulation of Munc18c expression by insulin, either directly or through different nuclear receptors and transcription factors.

As the amount of adipose tissue is very important in severe obesity, Munc18c from adipose tissue could play a relevant role in the regulation of insulin-stimulated exocytosis of GLUT4-containing vesicles. Despite the evidence, few data exist about Munc18c levels in the most extreme form of obesity. Given this situation, we analyzed Munc18c expression levels in visceral and subcutaneous adipose tissue from groups of lean controls, overweight, obese and morbidly obese patients and their association with insulin and different nuclear receptors related to lipid and carbohydrate metabolism in these tissues. We also undertook a prospective study of the association of Munc18c expression with the changes produced in morbidly obese patients after bariatric surgery.

## Materials and Methods

### Subjects

We evaluated 70 subjects distributed in two different cohorts. The first cohort included 23 nondiabetic morbidly obese patients (body mass index, (BMI) 57.4±5.2 Kg/m^2^). These morbidly obese patients included 11 with low insulin resistance (MO-L-IR) (homeostasis model assessment of insulin resistance index (HOMA-IR)<4.7) and 12 with high insulin resistance (MO-H-IR) (HOMA-IR>8) [27]–[29]. All the morbidly obese patients underwent biliopancreatic diversion (BPD) of Scopinaro, and were also studied 15 days after bariatric surgery. Patients were excluded if they had type 2 diabetes mellitus, cardiovascular disease, arthritis, acute inflammatory disease, infectious disease, or were receiving drugs that could alter the lipid profile or the metabolic parameters at the time of inclusion in the study. The weight of all the persons had been stable for at least one month before bariatric surgery and none had renal involvement. A second cohort included 12 non-obese lean subjects (BMI 22.6±1.9 kg/m^2^), 23 overweight subjects (BMI 27.2±1.2 Kg/m^2^) and 12 obese subjects (BMI 32.1±2.4 Kg/m^2^). These non-morbidly obese patients underwent laparoscopic surgery for hiatus hernia or cholelithiasis, with no alterations in lipid or glucose metabolism, and with a similar age and with the same selection criteria as those for the morbidly obese group. All subjects were of Caucasian origin and reported that their body weight had been stable for at least 3 months before the study. All participants gave their written informed consent and the study was reviewed and approved by the Ethics and Research Committee of Virgen de la Victoria Clinical University Hospital, Malaga, Spain.

### Laboratory Measurements

Blood samples from all subjects were collected after a 12-hour fast. The serum was separated and immediately frozen at –80°C. Serum biochemical parameters were measured in duplicate. Serum glucose, cholesterol, high density lipoprotein (HDL) cholesterol and triglycerides (Randox Laboratories Ltd., Antrim, UK) were measured by standard enzymatic methods. Adiponectin levels were measured by enzyme-linked immunosorbent assay (ELISA) kits (DRG Diagnostics, Marburg, Germany). The insulin was analyzed by an immunoradiometric assay (BioSource International, Camarillo, CA). The HOMA-IR was calculated from fasting insulin and glucose with the following equation: HOMA-IR = fasting insulin (µIU/mL)×fasting glucose (mol/L)/22.5. The percent change (Δ) of anthropometric and biochemical variables 15 days after bariatric surgery was calculated as (baseline variable - variable at 15 days)×100/baseline variable [30]. This point was chosen because during these 15 days, the morbidly obese patients are fed with the same type and quantity of liquid food.

### Adipose Tissue Samples

Visceral (VAT) and subcutaneous (SAT) adipose tissues were obtained during bariatric surgery in the morbidly obese patients and during laparoscopic surgery in the non-morbidly obese patients [28], [31]. The biopsy samples were washed in physiological saline and immediately frozen in liquid nitrogen. Biopsy samples were maintained at −80°C until analysis. Another SAT sample from the lean subjects was placed in phosphate buffered saline (PBS) supplemented with 5% bovine serum albumin (BSA) to perform adipose tissue explant cultures.

### Adipose Tissue Culture

Adipose tissue explants from 4 lean subjects were prepared by cutting samples into 5 mg portions, which were subsequently incubated for 30 min in PBS +5% BSA (3 ml/gr). After 30 seconds of centrifugation (400 g), samples were incubated in M199 medium (Life Technologies, Grand Island, NY) supplemented with 10% fetal bovine serum (FBS), 100 U/ml penicillin, and 100 µg/ml streptomycin. Explants were incubated in triplicate in the presence of liver X receptor-α (LXRα) agonist (T0901317) (10 µM) (Sigma-Aldrich, St. Louis, MO) and sterol regulatory element-binding protein-1c (SREBP-1c) inhibitor (Betulin) (6 µg/ml) [32] (Sigma-Aldrich, St. Louis, MO), in the presence or absence of insulin (1000 nM) (Actrapid®, Novo Nordisk A/S, Denmark), for 24 hours at 37°C. This dose of insulin has been used to analyze whether high hyperinsulinism can exert an effect on Munc18c expression [33]–[35]. Following these treatments, adipose tissue explants were collected and frozen in liquid nitrogen and stored at −80°C for further processing.

### RNA Extraction

Frozen VAT, SAT and culture explants were homogenized with an Ultra-Turrax 8 (Ika, Staufen, Germany). Total RNA was extracted by RNeasy lipid tissue midi kit (QIAGEN Science, Hilden, Germany), and total RNA was treated with 55U RNase-free deoxyribonuclease (QIAGEN Science, Hilden, Germany) following the manufacturer’s instructions. The purity of the RNA was determined by the absorbance260/absorbance280 ratio on a Nanodrop ND-1000 spectrophotometer (Thermo Fisher Scientific Inc. Waltham, MA). The integrity of total purified RNA was checked by denaturing agarose gel electrophoresis.

### Real-time Quantitative Polymerase Chain Reaction (PCR)

Total RNA was reverse transcribed to cDNA by using a high-capacity cDNA reverse transcription kit with RNase inhibitor (Applied Biosystems, Foster City, CA). The cDNA was used for quantitative real-time PCR with duplicates. We analyzed the relative baseline mRNA expression levels of Munc18c (Hs01029364_m1, RefSeq. NM_007269.2), LXRα (Hs00172885_m1, RefSeq. NM_001130102.1, NM_001130101.1 and NM_005693.2), SREBP-1c (Hs01088691_m1, RefSeq. NM_004176.3 and NM_001005291.1) and Peroxisome proliferator-activated receptor gamma (PPARγ) (Hs01115510_m1, Refseq. NM_015869.4). The cycle threshold (Ct) value for each sample was normalized with the expression of cyclophilin A (PPIA*)* (4326316E, RefSeq. NM_021130.3). The amplifications were performed using a MicroAmp® Optical 96-well reaction plate (Applied Biosystems, Foster City, CA) on an ABI 7500 Fast Real-Time PCR System (RT-qPCR) (Applied Biosystems, Foster City, CA). RT-qPCR reactions were carried out for all genes using specific TaqMan® Gene Expression Assays (Applied Biosystems, Foster City, CA). During PCR, the Ct values for each amplified product were determined using a threshold value of 0.1. SDS software 2.3 and RQ Manager 1.2 (Applied Biosystems, Foster City, CA) were used to analyze the results with the comparative Ct method (2^−ΔΔCt^). All data were expressed as an n-fold difference relative to the calibrator (a mixture of the SAT and VAT tissues was used as the calibrator sample).

### Statistical Analysis

The statistical analysis was done with Statistical Package for the Social Sciences (SPSS) (Version 11.5 for Windows; SPSS, Chicago, IL). Because most of the parameters analyzed do not have a normal distribution, we used non-parametric tests. Differences between two related variables were analyzed by the Wilcoxon test. Differences between more than two groups were compared using the Kruskal-Wallis test. The Spearman correlation coefficients were calculated to estimate the correlations between variables. Multiple linear regressions were used to determine the association between variables. Values were considered to be statistically significant when *P*≤0.05. The results are given as the mean ± standard deviation (SD).

## Results

### Anthropometric and Biochemical Characteristics

Table 1 summarizes the characteristics of the different groups of patients. The MO-H-IR patients had a higher weight, BMI, waist circumference, serum insulin and HOMA-IR than the MO-L-IR, lean, overweight and obese persons (Table 1).

**Table 1:** Anthropometric and biochemical variables in the lean, overweight, obese and morbidly obese persons classified according to their insulin resistance**.**

	Lean	Overweight	Obese	MO-L-IR	MO-H-IR
**Sex (male/female)**	6/6	14/9	7/5	5/6	6/6
**Age (years)**	39.6±12.6	57.1±15.0	57.4±12.8	42.4±11.1	37.5±10.1
**Weight (Kg)**	65.0±10.6^e^	74.7±10.2^d^	91.5±15.5^c^	135.8±30.6^b^	157.0±27.8^a^
**BMI (kg/m^2^)**	22.6±1.9^d^	27.3±1.4^c^	32.5±2.6^b^	52.4±9.4^a^	59.0±7.8^a^
**Waist (cm)**	80.0±9.5^e^	99.6±10.7^d^	114.5±10.7^c^	131.2±15.0^b^	149.5±17.1^a^
**Hip (cm)**	88.4±7.9^d^	93.7±11.8^c^	109.0±13.3^b^	152.0±17.4^a^	159.5±14.0^a^
**Glucose (mmol/L)**	4.63±0.61^b^	5.51±0.51^a^	5.61±0.45^a^	4.83±0.55^b^	5.51±0.53^a^
**Cholesterol (mmol/L)**	5.04±1.03	5.01±1.10	5.32±0.79	5.21±1.11	4.51±0.86
**Triglycerides (mmol/L)**	1.00±0.55^b^	1.29±0.77^a^	1.00±0.36^b^	1.18±0.66^a.b^	1.57±0.48^a^
**Insulin (µIU/ml)**	7.3±3.3^b^	5.2±3.6^b^	9.5±6.1^b^	10.3±3.6^b^	34.9±10.2^a^
**HOMA-IR**	1.48±0.58^b^	1.31±0.94^b^	2.48±1.61^b^	2.23±0.66^b^	8.51±2.03^a^
**Adiponectin (ng/ml)**	12.7±4.5	12.1±5.9	15.2±7.6	10.6±4.9	8.1±4.6

The results are given as the mean ± standard deviation. MO-L-IR: Morbid obesity with low insulin resistance; MO-H-IR: Morbid obesity with high insulin resistance; BMI: body mass index; HOMA-IR: homeostasis model assessment of insulin resistance index. Different letters indicate significant differences between the means of the different groups (P<0.05).

### Munc18c is Down-regulated in Adipose Tissue from Morbidly Obese Patients

**Figure 1:**
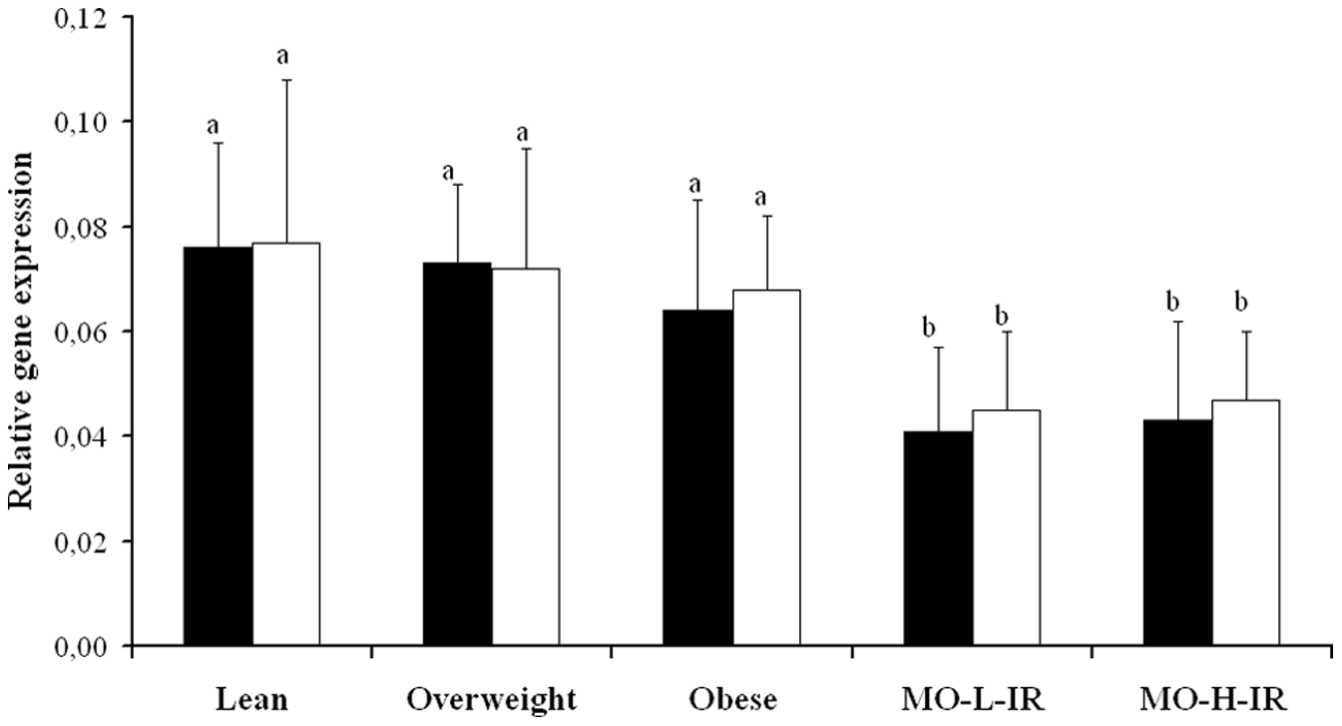
Munc18c gene expression in adipose tissue from the different groups of patients. The lean, overweight and obese persons had a significantly greater Munc18c gene expression in subcutaneous (SAT Munc18c) (□) and visceral (VAT Munc18c) adipose tissue (▪) than the morbidly obese cohort. MO-L-IR: morbidly obese persons with low insulin resistance. MO-H-IR: morbidly obese persons with high insulin resistance. The results are given as the mean ± standard deviation. Different letters indicate significant differences between the means of the different groups of subjects (P<0.05).

The lean, overweight and obese persons had a significantly greater Munc18c gene expression in VAT and SAT depots than the morbidly obese cohort (both MO-L-IR and MO-H-IR patients; *p*<0.001) (Figure 1). No significant differences were detected between the Munc18c gene expression regarding VAT and SAT depots in any of the study groups (Figure 1).

### Munc18c Expression Mainly Correlates with BMI

Munc18c gene expression levels in VAT and SAT correlated negatively with weight, BMI, and waist and hip circumferences (Table 2). Munc18c gene expression in VAT correlated negatively with glucose and positively with adiponectin (Table 2). Munc18c gene expression in SAT correlated negatively with insulin and HOMA-IR (Table 2). The expression of Munc18c in VAT and SAT did not correlate significantly with any of the other anthropometric and biochemical variables studied (data not shown).

**Table 2:** Bivariate correlations between Munc18c gene expression from subcutaneous (SAT) and visceral (VAT) adipose tissue gene expression and anthropometric and metabolic characteristics in the whole population.

	SAT MUNC-18c		VAT MUNC-18c	
	R	P	R	P
**Weight**	−0.402	0.018	−0.380	0.022
**BMI**	−0.411	0.016	−0.458	0.005
**Waist**	−0.374	0.032	−0.429	0.010
**Hip**	−0.453	0.008	−0.419	0.012
**Glucose**	Ns	Ns	−0.359	0.027
**Insulin**	−0.359	0.032	Ns	Ns
**HOMA-IR**	−0.349	0.037	Ns	Ns
**Adiponectin**	Ns	Ns	0.458	0.010

R: Spearman correlation coefficient. Ns: Not significant. BMI: body mass index; HOMA-IR: homeostasis model assessment of insulin resistance index.

In order to strengthen the independence of these associations as predictors of Munc18c gene expression, a multiple regression analysis model was constructed for each depot. In the VAT depot model, sex, age, BMI, waist and hip circumferences and adiponectin were selected as independent variables. VAT Munc18c gene expression was mainly predicted by the BMI (B = −0.001, *p* = 0.009; 95% confidence interval [CI] = −0.001–0.000). In SAT, no associations were found with different multiple regression analysis models.

### Munc18c Expression Correlates with Improvement in BMI and Insulin Resistance in Morbidly Obese Patients After Bariatric Surgery

Table 3 shows the differences in the anthropometric and biochemical variables between before the bariatric surgery and 15 days after the surgery. There was a significant improvement in the anthropometric and biochemical variables as a result of the bariatric surgery (Table 3). Munc18c gene expression in VAT correlated positively with the Δ-weight (R = 0.522, *p* = 0.045) and with the Δ-BMI (R = 0.522, *p* = 0.045). The Munc18c gene expression in SAT correlated negatively with the Δ-HOMA-IR (R = −0.708, *p* = 0.010). In a multiple linear regression model, the Δ-HOMA-IR was mainly predicted by the SAT Munc18c gene expression (R^2^ = 0.947) (B = −2148.4, *p* = 0.038; 95% CI = (−4072.7)−(−224.1)), adjusting for age, sex, Δ-BMI, Δ-waist and Δ-hip circumference. No significant associations were observed between the Munc18c gene expression in SAT or VAT and the evolution of the Δ-waist and Δ-hip circumferences and Δ-adiponectin during the prospective study (data not shown).

**Table 3:** Course of biochemical and anthropometric variables in the morbidly obese patients before and after bariatric surgery.

Variables	Beforesurgery	15 days after surgery	p
**Weight (Kg)**	153.0±30.1	142.2±23.0	0.002
**Waist (cm)**	142.7±18.1	137.5±13.9	0.040
**BMI (Kg/m^2^)**	56.2±8.7	52.2±6.9	0.002
**Glucose (mmol/L)**	5.10±0.66	5.32±0.57	0.195
**Cholesterol (mmol/L)**	4.85±1.05	4.11±1.20	0.048
**Triglycerides (mmol/L)**	1.50±0.88	2.00±0.70	0.057
**Insulin (µIU/ml)**	22.5±13.1	14.3±5.9	0.005
**HOMA-IR**	5.72±3.21	3.67±1.55	0.011
**Adiponectin (ng/ml)**	8.77±4.34	7.65±3.82	0.141

The results are given as the mean ± standard deviation. BMI: body mass index. HOMA-IR: homeostasis model assessment of insulin resistance index.

### Munc18c Expression Correlates with Different Nuclear Receptors

Associations between Munc18c gene expression with different genes related to lipid and carbohydrate metabolism were explored in VAT and SAT. In VAT, Munc18c gene expression levels did not correlate with any of genes analyzed (LXRα, SREBP-1c and PPARγ) (data not shown). In SAT, Munc18c gene expression correlated significantly and negatively with SREBP-1c (r = −0.626, p = 0.017) and LXRα (r = −0.590, p = 0.026), but not with PPARγ.expression levels (r = 0.114, p = 0.808).

### Insulin Down-regulates Munc18c Expression *in vitro*

**Figure 2:**
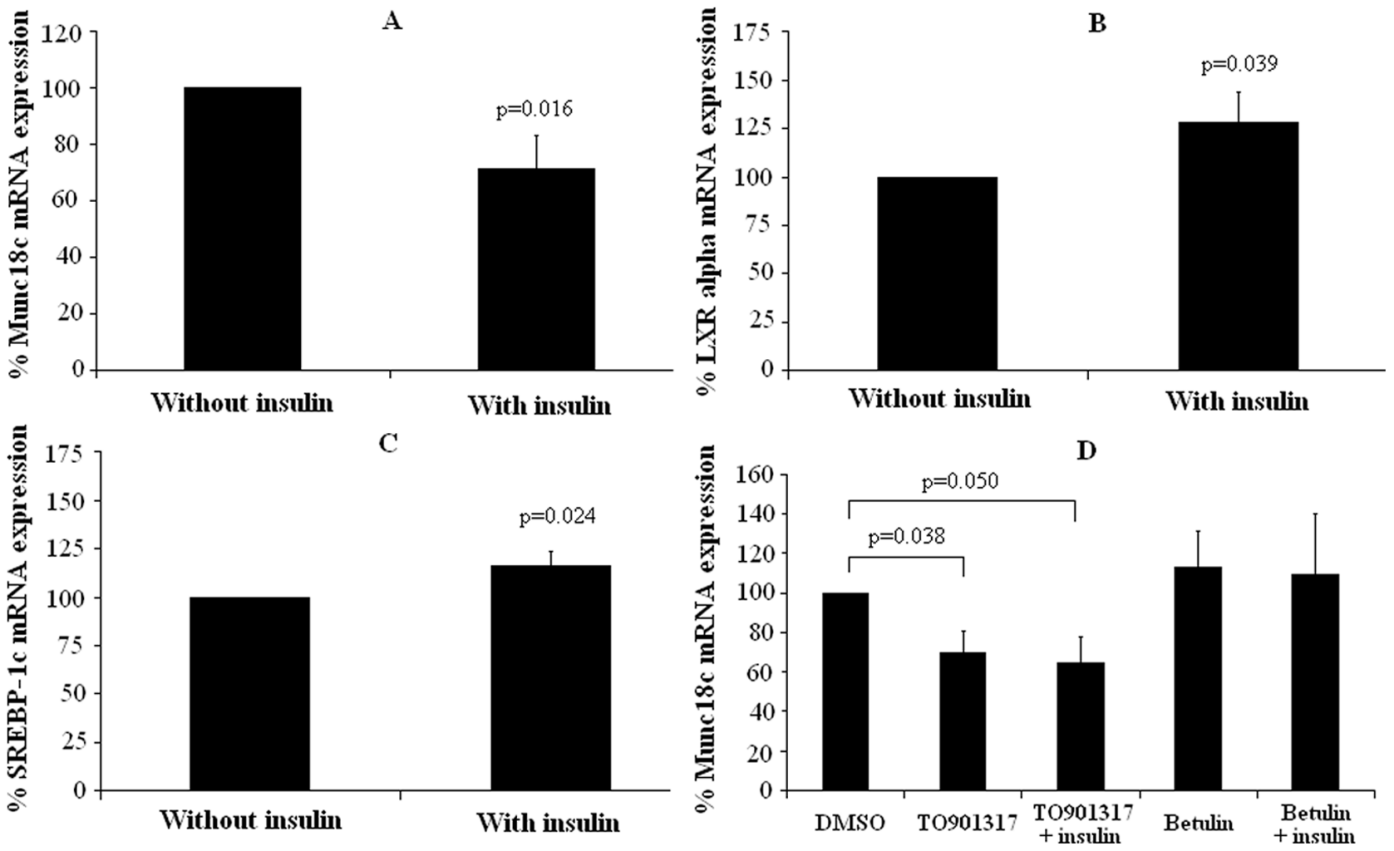
Gene expression in subcutaneous adipose tissue (SAT) explants (n = 4) incubated with different compounds. (A) Munc18c gene expression in SAT explants incubated without or with insulin (1000 nM). (B) Liver X receptor-alpha (LXRα) gene expression in SAT explants incubated without or with insulin (1000 nM). (C) Sterol regulatory element-binding protein-1c (SREBP-1c) gene expression in SAT explants incubated without or with insulin (1000 nM). (D) Munc18c gene expression in SAT explants incubated in the presence of LXRα agonist (T0901317) (10 µM) and SREBP-1c inhibitor (Betulin) (6 µg/ml), in the presence or absence of insulin (1000 nM). DMSO: Dimethyl sulfoxide. The results are given as the mean ± SEM.

Given the significant correlation found between insulin and Munc18c gene expression in SAT, we checked whether co-incubation with insulin could modify the Munc18c gene expression. Accordingly, SAT explants from 4 lean subjects were incubated without or with insulin (1000 nM). After 24 hours of incubation, a significant down-regulation of Munc18c gene expression was observed (p = 0.016) (Figure 2a). However, a significant up-regulation of LXRα (p = 0.039) (Figure 2b) and SREBP-1c expression (p = 0.024) (Figure 2c) was also found.

To determine whether Munc18c is related to LXRα and SREBP-1c, we made different experiments with 10 µM T0901317, a LXRα agonist, and with 6 µg/ml betulin, a SREBP-1c inhibitor, both with and without 1000 nM insulin. Figure 2d shows that Munc18c gene expression was significantly decreased when SAT explants were incubated with T0901317, both without (p = 0.038) and with insulin (p = 0.050). However, Munc18c gene expression was not affected when SAT explants were incubated with betulin, either without (p = 0.806) or with insulin (p = 0.504) (Figure 2d).

## Discussion

The results show that Munc18c gene expression in human VAT and SAT were down-regulated in morbidly obese patients. However, human data are very scarce regarding adipose tissue expression. Also, SAT Munc18c gene expression was inversely associated with insulin. Moreover, insulin down-regulated the Munc18c gene expression in SAT culture.

A new finding of this study is the inverse association between Munc18c gene expression in adipose tissue and many clinical variables associated with a poorly metabolic profile, such as weight, BMI, and waist and hip circumferences. Although these bivariate associations were observed both in SAT and VAT depots, after controlling for confounding variables, BMI was the only factor determining Munc18c gene expression in VAT, with no effect on SAT. We are aware of the observational design of the study and that no cause-consequence events may be inferred. However, in the prospective study in the morbidly obese cohort, basal Munc18c VAT gene expression levels were inversely associated with weight loss at the short-term evaluation after bariatric procedure. This finding tempted us to speculate a role of VAT Munc18c in the early metabolic events that occur in the few days after surgery in morbidly obese patients.

Munc18c is part of the insulin-signalling steps in GLUT4 vesicle exocytosis. Reduced protein and/or mRNA levels of Munc18c is reported in obese and type 2 diabetic human subjects as well as in obese rodent and diabetic models [36]. The inverse association with circulating levels of glucose and insulin may link Munc18c gene expression in adipose tissue with the effects of insulin. Despite the lack of differences in the basal study between the high and low insulin resistant morbidly obese patients regarding Munc18c gene expression, the prospective analysis in the surgically treated cohort showed that the HOMA-IR index improvement after bariatric surgery was associated with SAT Munc18c gene expression. This suggests that Munc18c in SAT may be involved in the early improvement of the morbidly obese subjects after bariatric surgery. This association opens a new path to understand the *switch on* in glucose metabolism that takes place in the early days after bariatric surgery, before the appearance of a significant weight loss.

Emerging evidence suggests that Munc18c can be regulated by insulin. Insulin regulates various aspects of GLUT4 exocytosis. Stimulation of 3T3-L1 adipocytes with insulin promotes tyrosine phosphorylation of Munc18c [24]–[26], a modification that occurs concomitantly with an observed dissociation of the sintaxin-4/Munc18c complex [23]. Our *in vitro* study in SAT explants argues in favour of a modulation by insulin of Munc18c gene expression.

**Figure 3:**
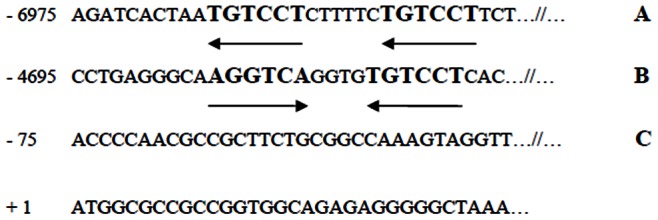
Selected sequence of the human Munc18c 5′-flanking region derived from the NCBI Reference Sequence NC_000001.10. The sequence of the human Munc18c 5′-flanking region (∼7 kb) is shown with the LXR response elements (LXREs) denoted by arrows. These LXREs contain a (A) direct repeats (DRs) of the AGGACA half-sites spaced by 6 bp, and (B) inverted repeats (IRs) of AGGTCA and AGGACA half-sites spaced by 4 bp. (C) The putative transcription start site at −75 is shown.

To corroborate the *in vivo* correlations between LXRα and SREBP-1c with Munc18c in SAT, and whether the *in vitro* effect of insulin may be mediated by LXRα and SREBP-1c, we employed different preliminary *in vitro* experiments with SAT explants. It is known that insulin activates the SREBP-1c promoter, primarily by increasing the activity of LXR [37], [38]. The above-mentioned experiment showed that insulin up-regulates the LXRα and SREBP-1c expression levels. This finding is in agreement with other studies, in which the expression of LXRα and SREBP-1c is regulated by insulin [37]–[39]. To date, little is known about the Munc18c regulation by different nuclear receptors and transcription factors. Our *in vitro* experiment showed that Munc18c expression in SAT is down-regulated when SAT is incubated with a LXRα agonist. It is known that LXR ligands may inhibit the transcription of certain genes through different mechanisms [40], [41]. However, incubation with the LXRα agonist and insulin did not produce a greater decrease in Munc18c expression. This result may suggest that insulin can exert its effects on SAT Munc18c through LXRα. After activation, LXR binds DNA as obligate heterodimers with retinoid X receptors (RXRs). The RXR/LXR heterodimer binds with high affinity in the promoter regions of LXR target genes to the LXR response element (LXRE), a DNA sequence comprised of two direct repeats of the same AGGTCA half-sites separated with different spacings (DR) [42]–[44]. We found different LXREs in the Munc18c promoter (Figure 3), although this information is only informative. Naturally-occurring hormone response elements can contain half-sites that diverge significantly from the AGGTCA consensus sequences, like AGGACA. However, only a small percent of the LXR:RXR binding sites contain well-defined consensus sequences or half-site spacings, indicating that these heterodimers can bind to a variety of half-site spacings [44]–[46]. Changing the spacing in these natural elements had only modest effects on the ability of their receptors to bind to these sequences. This *in vitro* study is a preliminary experiment to examine whether LXR may be involved in the regulation of Munc18c gene expression. Further functional studies will be required to determine the involvement of LXRE in Munc18c expression. It is also known that the effect of the LXRα agonist can be partially mediated by the increased expression of SREBP-1c [43]. The incubation of SAT with a SREBP-1c inhibitor or with insulin plus a SREBP-1c inhibitor prevented the decrease found in Munc18c gene expression when SAT was incubated only with insulin. The results, together the LXREs in the Munc18c promoter, suggest that the effect of insulin on the Munc18c gene expression in cultured SAT may be through LXRα and SREBP-1c.

There is a wealth of evidence implicating Munc18c in the control of insulin-stimulated GLUT4 translocation to the plasma membrane [15]–[17], [23], [26], [47]–[49]. We suggest that the decrease in Munc18c expression found in our study in the morbidly obese subjects may be a compensatory mechanism to favour GLUT4 translocation. However, other proteins may also be involved. The Munc18c protein is known to be regulated by different mechanisms. However, this is the first study to show that insulin can produce a decrease in SAT Munc18c gene expression. A precise characterization of the mechanisms by which Munc18c is regulated will allow us to better understand the trafficking itinerary of GLUT4.

In conclusion, our data demonstrate for the first time that the level of Munc18c gene expression in human adipose tissue is down-regulated in morbid obesity. We found that Munc18c gene expression was associated with the short-term metabolic improvement after bariatric surgery. However, the data reported in this study are only descriptive and only show an association. Also, Munc18c gene expression was inversely associated with insulin in SAT. To our knowledge, the present study is the first to report the effects of insulin on SAT Munc18c gene expression in an *in vitro* model. As our preliminary experiment shows, this effect may be through LXRα and SREBP-1c. This observation clearly warrants further functional studies to show the precise mechanism involved in the regulation of Munc18c gene expression.

## References

[1] Libby P, Ridker PM, Maseri A (2002) Inflammation and atherosclerosis. Circulation 105: 1135–1143. Review. 10.1161/hc0902.104353 11877368

[2] James DE, Piper RC, Slot JW (1994) Insulin stimulation of GLUT-4 translocation: a model for regulated recycling. Trends Cell Biol 4: 120–126. 14731734 10.1016/0962-8924(94)90066-3

[3] Czech MP (1995) Molecular actions of insulin on glucose transport. Annu Rev Nutr 15: 441–471. Review. 10.1146/annurev.nu.15.070195.002301 8527229

[4] Klip A, Tsakiridis T, Marette A, Ortiz PA (1994) Regulation of expression of glucose transporters by glucose: a review of studies in vivo and in cell cultures. FASEB J 8: 43–53. Review. 10.1096/fasebj.8.1.8299889 8299889

[5] Thurmond DC, Pessin JE (2001) Molecular machinery involved in the insulin regulated fusion of GLUT4-containing vesicles with the plasma membrane. Mol Membr Biol 18: 237–245. Review. 10.1080/09687680110082400 11780752

[6] Pessin JE, Saltiel AR (2000) Signaling pathways in insulin action: molecular targets of insulin resistance. J Clin Invest 106: 165–169. Review. 10.1172/JCI10582 PMC314316 10903329

[7] Foster LJ, Klip A (2000) Mechanism and regulation of GLUT-4 vesicle fusion in muscle and fat cells. Am J Physiol Cell Physiol 279: C877–C890. Review. 10.1152/ajpcell.2000.279.4.C877 11003568

[8] Kawanishi M, Tamori Y, Okazawa H, Araki S, Shinoda H, et al (2000) Role of SNAP23 in insulin-induced translocation of GLUT4 in 3T3–L1 adipocytes: mediation of complex formation between syntaxin4 and VAMP2. J Biol Chem 275: 8240–7. 10713150 10.1074/jbc.275.11.8240

[9] Volchuk A, Wang Q, Ewart HS, Liu Z, He L, et al (1996) Syntaxin 4 in 3T3–L1 adipocytes: regulation by insulin and participation in insulindependent glucose transport. Mol Biol Cell 7: 1075–82. 8862521 10.1091/mbc.7.7.1075 PMC275959

[10] Yang C, Coker KJ, Kim K, Mora S, Thurmond DC, et al (2001) Syntaxin 4 heterozygous knockout mice develop muscle insulin resistance. J Clin Invest 107: 1311–8. 11375421 10.1172/JCI12274 PMC209300

[11] Tellam JT, Macaulay SL, McIntosh S, Hewish DR, Ward CW, et al (1997) Characterization of Munc-18c and syntaxin-4 in 3T3-L1 adipocytes. Putative role in insulin-dependent movement of GLUT-4. J Biol Chem 272: 6179–86. 9045631 10.1074/jbc.272.10.6179

[12] Hata Y, Sudhof TC (1995) A novel ubiquitous form of Munc-18 interacts with multiple syntaxins. Use of the yeast two-hybrid system to study interactions between proteins involved in membrane traffic. J Biol Chem 270: 13022–8. 7768895 10.1074/jbc.270.22.13022

[13] Pevsner J, Hsu SC, Scheller RH (1994) n-Sec1: a neural-specific syntaxin-binding protein. Proc Natl Acad Sci USA 91: 1445–9. 8108429 10.1073/pnas.91.4.1445 PMC43176

[14] Garcia EP, Gatti E, Butler M, Burton J, De Camilli P (1994) A rat brain Sec1 homologue related to Rop and UNC18 interacts with syntaxin. Proc Natl Acad Sci USA 91: 2003–7. 8134339 10.1073/pnas.91.6.2003 PMC43297

[15] Tamori Y, Kawanishi M, Niki T, Shinoda H, Araki S, et al (1998) Inhibition of insulin-induced GLUT4 translocation by Munc18c through interaction with syntaxin4 in 3T3-L1 adipocytes. J Biol Chem 273: 19740–6. 9677404 10.1074/jbc.273.31.19740

[16] Thurmond DC, Ceresa BP, Okada S, Elmendorf JS, Coker K, et al (1998) Regulation of insulin-stimulated GLUT4 translocation by Munc18c in 3T3L1 adipocytes. J Biol Chem 273: 33876–83. 9837979 10.1074/jbc.273.50.33876

[17] Thurmond DC, Kanzaki M, Khan AH, Pessin JE (2000) Munc18c function is required for insulin-stimulated plasma membrane fusion of GLUT4 and insulin-responsive amino peptidase storage vesicles. Mol Cell Biol 20: 379–88. 10594040 10.1128/mcb.20.1.379-388.2000 PMC85093

[18] Hosono R, Hekimi S, Kamiya Y, Sassa T, Murakami S, et al (1992) The unc-18 gene encodes a novel protein affecting the kinetics of acetylcholine metabolism in the nematode Caenorhabditis elegans. J Neurochem 58: 1517–25. 1347782 10.1111/j.1471-4159.1992.tb11373.x

[19] Harrison SD, Broadie K, van de Goor J, Rubin GM (1994) Mutations in the Drosophila Rop gene suggest a function in general secretion and synaptic transmission. Neuron 13: 555–66. 7917291 10.1016/0896-6273(94)90025-6

[20] Jahn R, Südhof TC (1999) Membrane fusion and exocytosis. Annu Rev Biochem 68: 863–911. Review. 10.1146/annurev.biochem.68.1.863 10872468

[21] Spurlin BA, Thomas RM, Nevins AK, Kim HJ, Kim YJ, et al (2003) Insulin resistance in tetracycline-repressible Munc18c transgenic mice. Diabetes 52: 1910–7. 12882905 10.2337/diabetes.52.8.1910

[22] Oh E, Spurlin BA, Pessin JE, Thurmond DC (2005) Munc18c heterozygous knockout mice display increased susceptibility for severe glucose intolerance. Diabetes 54: 638–47. 15734838 10.2337/diabetes.54.3.638

[23] Umahara M, Okada S, Yamada E, Saito T, Ohshima K, et al (2008) Tyrosine phosphorylation of Munc18c regulates platelet-derived growth factor-stimulated glucose transporter 4 translocation in 3T3L1 adipocytes. Endocrinology 149: 40–9. 17916632 10.1210/en.2006-1549

[24] Jewell JL, Oh E, Ramalingam L, Kalwat MA, Tagliabracci VS, et al (2011) Munc18c phosphorylation by the insulin receptor links cell signaling directly to SNARE exocytosis. J Cell Biol 193: 185–99. 21444687 10.1083/jcb.201007176 PMC3082181

[25] Schmelzle K, Kane S, Gridley S, Lienhard GE, White FM (2006) Temporal dynamics of tyrosine phosphorylation in insulin signaling. Diabetes 55: 2171–9. 16873679 10.2337/db06-0148

[26] Aran V, Bryant NJ, Gould GW (2011) Tyrosine phosphorylation of Munc18c on residue 521 abrogates binding to Syntaxin 4. BMC Biochem 12: 19. 21548926 10.1186/1471-2091-12-19 PMC3103433

[27] Garcia-Fuentes E, Murri M, Garrido-Sanchez L, Garcia-Serrano S, García-Almeida JM, et al (2010) PPARγ expression after a high-fat meal is associated with plasma superoxide dismutase activity in morbidly obese persons. Obesity (Silver Spring) 18: 952–8. 19816416 10.1038/oby.2009.314

[28] Tinahones FJ, Garrido-Sanchez L, Miranda M, García-Almeida JM, Macias-Gonzalez M, et al (2010) Obesity and insulin resistance-related changes in the expression of lipogenic and lipolytic genes in morbidly obese subjects. Obes Surg 20: 1559–67. 20512427 10.1007/s11695-010-0194-z

[29] Tinahones FJ, Murri-Pierri M, Garrido-Sánchez L, García-Almeida JM, García-Serrano S, et al (2009) Oxidative stress in severely obese persons is greater in those with insulin resistance. Obesity (Silver Spring) 17: 240–6. 19023278 10.1038/oby.2008.536

[30] Garrido-Sanchez L, Murri M, Rivas-Becerra J, Ocaña-Wilhelmi L, Cohen RV, et al (2012) Bypass of the duodenum improves insulin resistance much more rapidly than sleeve gastrectomy. Surg Obes Relat Dis 8: 145–50. 21570362 10.1016/j.soard.2011.03.010

[31] García-Serrano S, Moreno-Santos I, Garrido-Sánchez L, Gutierrez-Repiso C, García-Almeida JM, et al (2010) Stearoyl-CoA desaturase-1 is associated with insulin resistance in morbidly obese subjects. Mol Med 17: 273–80. 21060977 10.2119/molmed.2010.00078 PMC3060976

[32] Tang JJ, Li JG, Qi W, Qiu WW, Li PS, et al (2011) Inhibition of SREBP by a small molecule, betulin, improves hyperlipidemia and insulin resistance and reduces atherosclerotic plaques. Cell Metab 13: 44–56. 21195348 10.1016/j.cmet.2010.12.004

[33] Hugo ER, Borcherding DC, Gersin KS, Loftus J, Ben-Jonathan N (2008) Prolactin Release by Adipose Explants, Primary Adipocytes, and LS14 Adipocytes. J Clin Endocrinol Metab 93: 4006–12. 18647802 10.1210/jc.2008-1172 PMC2579649

[34] Harte AL, McTernan PG, McTernan CL, Crocker J, Starcynski J, et al (2003) Insulin increases angiotensinogen expression in human abdominal subcutaneous adipocytes. Diabetes Obes Metab 5: 462–7. 14617233 10.1046/j.1463-1326.2003.00274.x

[35] Yu X, Shen N, Zhang ML, Pan FY, Wang C, et al (2011) Egr-1 decreases adipocyte insulin sensitivity by tilting PI3K/Akt and MAPK signal balance in mice. EMBO J 30: 3754–65. 21829168 10.1038/emboj.2011.277 PMC3173797

[36] Bergman BC, Cornier MA, Horton TJ, Bessesen DH, Eckel RH (2008) Skeletal muscle munc18c and syntaxin 4 in human obesity. Nutr Metab (Lond) 5: 21. 18652694 10.1186/1743-7075-5-21 PMC2515313

[37] Chen G, Liang G, Ou J, Goldstein JL, Brown MS (2004) Central role for liver X receptor in insulin-mediated activation of Srebp-1c transcription and stimulation of fatty acid synthesis in liver. Proc Natl Acad Sci USA 101: 11245–50. 15266058 10.1073/pnas.0404297101 PMC509189

[38] Dif N, Euthine V, Gonnet E, Laville M, Vidal H, et al (2006) Insulin activates human sterol-regulatory-element-binding protein-1c (SREBP-1c) promoter through SRE motifs. Biochem J 400: 179–88. 16831124 10.1042/BJ20060499 PMC1635455

[39] Tobin KA, Ulven SM, Schuster GU, Steineger HH, Andresen SM, et al (2002) Liver X receptors as insulin-mediating factors in fatty acid and cholesterol biosynthesis. J Biol Chem Mar 277: 10691–7. 10.1074/jbc.M109771200 11781314

[40] Wójcicka G, Jamroz-Wiśniewska A, Horoszewicz K, Bełtowski J (2007) Liver X receptors (LXRs). Part I: structure, function, regulation of activity, and role in lipid metabolism. Postepy Hig Med Dosw (Online) 61: 760–85. Review. 18063918

[41] Kuipers I, Li J, Vreeswijk-Baudoin I, Koster J, van der Harst P, et al (2010) Activation of liver X receptors with T0901317 attenuates cardiac hypertrophy in vivo. Eur J Heart Fail 12: 1042–50. 20587624 10.1093/eurjhf/hfq109

[42] Phan TQ, Jow MM, Privalsky ML (2010) DNA recognition by thyroid hormone and retinoic acid receptors: 3,4,5 rule modified. Mol Cell Endocrinol 319: 88–98. 19945505 10.1016/j.mce.2009.11.010 PMC3270409

[43] Repa JJ, Liang G, Ou J, Bashmakov Y, Lobaccaro JM, et al (2000) Regulation of mouse sterol regulatory element-binding protein-1c gene (SREBP-1c) by oxysterol receptors, LXRa and LXRb. Genes Dev 14: 2819–30. 11090130 10.1101/gad.844900 PMC317055

[44] Boergesen M, Pedersen TÅ, Gross B, van Heeringen SJ, Hagenbeek D, et al (2012) Genome-wide profiling of liver X receptor, retinoid X receptor, and peroxisome proliferator-activated receptor α in mouse liver reveals extensive sharing of binding sites. Mol Cell Biol 32: 852–67. 22158963 10.1128/MCB.06175-11 PMC3272984

[45] Pehkonen P, Welter-Stahl L, Diwo J, Ryynänen J, Wienecke-Baldacchino A, et al (2012) Genome-wide landscape of liver X receptor chromatin binding and gene regulation in human macrophages. BMC Genomics 13: 50. 22292898 10.1186/1471-2164-13-50 PMC3295715

[46] Moutier E, Ye T, Choukrallah MA, Urban S, Osz J, et al (2012) Retinoic acid receptors recognize the mouse genome through binding elements with diverse spacing and topology. J Biol Chem 287: 26328–41. 22661711 10.1074/jbc.M112.361790 PMC3406717

[47] Macaulay SL, Grusovin J, Stoichevska V, Ryan JM, Castelli LA, et al (2002) Cellular munc18c levels can modulate glucose transport rate and GLUT4 translocation in 3T3L1 cells. FEBS Lett 528: 154–60. 12297296 10.1016/s0014-5793(02)03279-9

[48] Khan AH, Thurmond DC, Yang C, Ceresa BP, Sigmund CD, et al (2001) Munc18c regulates insulin-stimulated glut4 translocation to the transverse tubules in skeletal muscle. J Biol Chem 276: 4063–9. 11054418 10.1074/jbc.M007419200 PMC5540311

[49] Spurlin BA, Park SY, Nevins AK, Kim JK, Thurmond DC (2004) Syntaxin 4 transgenic mice exhibit enhanced insulin-mediated glucose uptake in skeletal muscle. Diabetes 53: 2223–31. 15331531 10.2337/diabetes.53.9.2223

